# Epithelial-Mesenchymal Transition: A Potential Therapeutic Goal for Prevention of Wound Fibrosis?

**Published:** 2006-11-30

**Authors:** Ramin Mostofi Zadeh Farahani

**Affiliations:** School of Dentistry, Tabriz University of Medical Sciences, Tabriz, Iran

The skin is a composite structure composed of a superficial epidermis and an underlying dermis. Wounding of this structure with resultant functional and anatomical disintegration leads to a cascade of events directed at the restoration of these features. However, maintenance of anatomical integrity has a higher priority from the standpoint of preservation of homeostatic equilibrium. The uncoupling of anatomical and functional renovation due to accelerated wound closure without proper regeneration and spatial organization of the underlying cellular/extracellular assembly leads to scarring and loss of function. Epithelialization and contraction are the major mechanisms acting to minimize the exposed wound surface.^1^ Disconnection of these two procedures, especially for extensive superficial wounds, for example burns, may prove useful; the potential delay between these procedures, while keeping the epithelialization at an optimum level, provides sufficient time for spatial organization of the underlying matrix and thereby prevents loss of function.

Fibroblasts and epithelial cells are major regulatory elements of wound contraction and epithelialization, respectively. Fibroblasts contribute to contraction directly by producing contractile forces^2^ and indirectly via differentiating into myofibroblasts.^3^ The epithelium, once considered to be terminally differentiated, has the ability to differentiate into fibroblasts.^4^,^5^ The occurrence of epithelial-mesenchymal transition (EMT) following epithelial stress such as inflammation or wound has been documented.^4^–^7^ Local expression of TGF-β, EGF, IGF-II, or FGF-2 facilitates EMT by binding epithelial receptors with ligand-inducible intrinsic kinase activity.^8^–^11^ While during somitogenesis, mesenchymal-epithelial transition happens,^12^ this transformation has not been investigated in the wound milieu. Iwano et al^5^ suggest that the majority of local fibroblasts develop following EMT from epithelium. Manipulation of cell fate pathways of the epithelium toward reepithelialization while diminishing EMT may serve to prevent the aforementioned excessive fibrosis and the resultant anatomical-functional uncoupling. TGF-β exerts an inhibitory effect on the proliferating epithelium^13^ and the EGF/IGF assembly synergistically enhances it.^14^ It has been shown that TGF-β exerts an inhibitory effect on EGF.^15^,^16^ Therefore, although speculative, it seems that altering the ratio of TGF-β/EGF in favor of EGF may help reduce EMT and consequent fibrosis and scarring. This hypothesis is further supported by the finding that TGF-β1–null mice show enhanced wound healing, with narrower, scabless wounds. The rate of reepithelialization in the knock-out mice increased such that, 3 days postwounding, wounds were 90% covered with the neoepidermis compared with only 22% of the wound surface in controls.^17^ Furthermore, while neutralizing antibodies to TGF-β1 reduced scarring,^18^ treatment of fetal wounds with different concentrations of TGF-β1 caused marked scarring of these wounds,^19^ suggesting a pivotal role for TGF-β in the scarring. The enhanced epithelialization and diminished scarring—that is, disconnection of these two procedures—and the resultant anatomical-functional uncoupling are in agreement with the proposed hypothesis.

This prospective modality may be useful for wounds where excessive contraction and fibrosis is probable, for example, extensive burns. Future research may be directed toward testing the hypothesis via EMT markers and also developing therapeutic strategies for regulation of the TGF/EGF ratio.
